# Nonobstructive Acute Renal Failure with a Large Solitary Fibroid

**DOI:** 10.1155/2016/4039890

**Published:** 2016-06-07

**Authors:** Rayan Elkattah, Zineb Mashak, Blakele Bakker, Shanti Mohling, Ali Yilmaz, Stephen DePasquale, Todd Boren

**Affiliations:** ^1^Department of Obstetrics and Gynecology, University of Tennessee Chattanooga College of Medicine, Chattanooga, TN 37403, USA; ^2^Gülhane Military Medical Academy Haydarpasa Training Hospital, Department of Obstetrics and Gynecology, Selimiye Mahallesi, Tibbiye Caddesi, 34668 Istanbul, Turkey; ^3^Division of Gynecology Oncology, University of Tennessee Chattanooga College of Medicine, Chattanooga, TN 37403, USA

## Abstract

A 38-year-old African American woman presenting with acute abdominal pain and nonobstructive renal failure was found to have an enlarged fibroid uterus. A differential for sepsis was considered. Lab evaluation revealed an elevated creatinine and myoglobin level at 3.9 mg/dL and 2140 ng/mL, respectively. Ongoing hemodynamic instability mandated surgery for acute abdomen. A 25 cm fibroid uterus was extirpated through a total abdominal hysterectomy. Immediate improvement of acute nephropathy mirrored the postoperative decline in serum myoglobin levels. Myoglobinemia from a massive degenerating fibroid is associated with nonobstructive acute renal failure.

## 1. Introduction

Uterine fibroids constitute the most common tumor in women of reproductive age [1]. Significant morbidity secondary to fibroids is a rare event; however acute complications from fibroids may include thromboembolic events, acute torsion of pedunculated fibroids, acute abdominal pain, vaginal bleeding, intra-abdominal bleeding, acute urinary retention, and renal failure. Uterine fibroids are associated with obstructive renal failure as they can physically compress the ureters, leading to acute urinary retention and postrenal nephropathy.

Myoglobin is a major component of skeletal muscle tissue and constitutes up to 0.2–0.6 mg/g of uterine smooth muscle weight [2]. The inflammatory response that occurs with fibroid degeneration is associated with acute onset of pain, fever, and vaginal bleeding. The potential for myoglobin to leak out from a degenerating fibroid into circulation may lead to elevations in serum myoglobin levels. As the diagnosis of renal failure with fibroids is one of exclusion, we report the case of acute renal failure associated with a large degenerating fibroid and abnormal myoglobinemia.

## 2. Case

A 38-year-old G_2_P_2_ African American woman presented with 3-hour duration of severe abdominal pain, nausea, and vomiting. Her pain was acute in onset and constant in nature, radiated to both groins, and was progressively worsening. She denied any musculoskeletal pain. Her past medical history was significant for hypertension, fibroid uterus, and a cholecystectomy. She had worsening menorrhagia over the past two months. Her last gynecologic evaluation was three years prior to presentation. She reported tobacco use and denied alcohol or drug consumption. Physical examination revealed diffuse rebound tenderness and a palpable abdominal mass. Initial vital signs were as follows: temperature of 38.6°C, heart rate of 122 beats per minute, blood pressure of 48/37, and respiratory rate of 31 breaths per minute. Oxygen saturation was 88% and increased to 94% on 2 liters of nasal oxygen.

**Figure 1:**
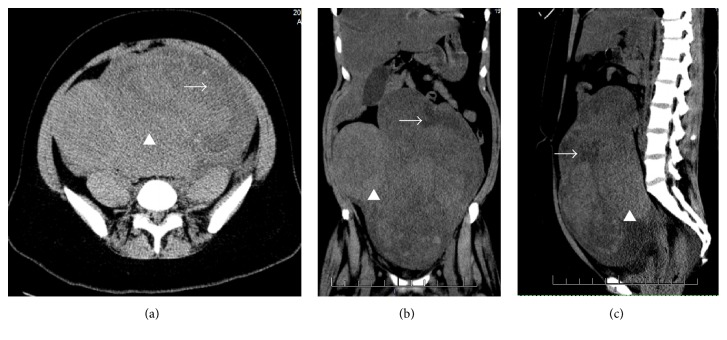
Computed tomography scan of the abdomen and pelvis without contrast showing the pelvic mass with corresponding solid (arrowhead) and cystic components (arrow). Axial (a), coronal (b), and sagittal (c) views.

Biochemical testing showed an elevated white blood cell count (13.5 K/*μ*L), significant neutrophilia (94.8%), an elevatedcreatinine (3.8 mg/dL), low hemoglobin (8.9 g/dL), low potassium (3.2 mEq/L), and an elevated myoglobin (2140 ng/mL, normal range: 0–101 ng/mL). A cardiac and skeletal muscle enzyme panel was normal. Baseline creatinine from a prior admission was normal (0.6 mg/dL). Urinalysis showed trace blood and amorphous sediment. A computed tomography scan of the abdomen and pelvis without contrast showed a 24 cm × 13 cm × 13 cm complex mass occupying the pelvis and extending to the upper abdomen (Figure 1). Both kidneys appeared normal in size without perinephric inflammation, hydronephrosis, or hydroureter. Renal sonography revealed normal echotexture and ureteral caliber.

The working diagnosis was acute septic shock with associated acute renal failure. The patient had nonanion gap metabolic acidosis with a lactic acid level of 4.5 mmol/L (normal 0.4–2 mmol/L) and a pH of 7.40 with a compensatory decrease in P_CO_2__ secondary to hyperventilation. Blood and urine cultures were obtained and the patient was started on intravenous fluid resuscitation, a bicarbonate drip, and broad-spectrum antibiotics. A norepinephrine infusion was used to maintain a normotensive state. The pelvic mass was thought to be the source of the patient's abdominal pain and represented a degenerating fibroid that was contributing to her critical presentation with worsening inflammation and myoglobinemia. Relevant tumor markers including CEA, AFP, CA-125, and inhibins A and B were normal.

The patient's overall status continued to worsen the following day. She became obtunded with loss of consciousness, in sinus tachycardia, and hypotensive despite all aforementioned measures. Endotracheal intubation then followed. Furthermore, her renal function continued to deteriorate (creatinine of 7 mg/dL). Urine and blood cultures had still not yielded any bacterial growth. Continuous renal replacement therapy (CRRT) was initiated and the decision to perform an exploratory laparotomy and remove the pelvic mass followed, as it was attributed to the patient's worsening critical state.

A large solitary fibroid was found within the left broad ligament that was densely adherent and contiguous with the uterus. The uterus and adnexal structures appeared significantly devitalized and edematous and were displacing the ureters without evidence of hydroureter. The bowels appeared normal. A total abdominal hysterectomy with bilateral salpingo-oophorectomy was performed with an estimated blood loss of 150 mL. The patient remained intubated and returned to the surgical intensive care unit. The postoperative period was complicated by atrial fibrillation that responded to intravenous amiodarone in addition to anemia (Hg 6.7 g/dL) requiring blood transfusion.

**Figure 2:**
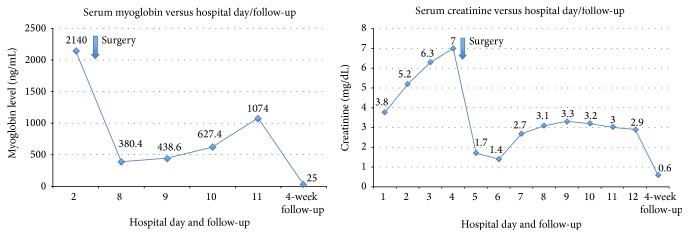
Serum myoglobin and creatinine levels during the patient's hospitalization and on the 4-week follow-up.

**Figure 3:**
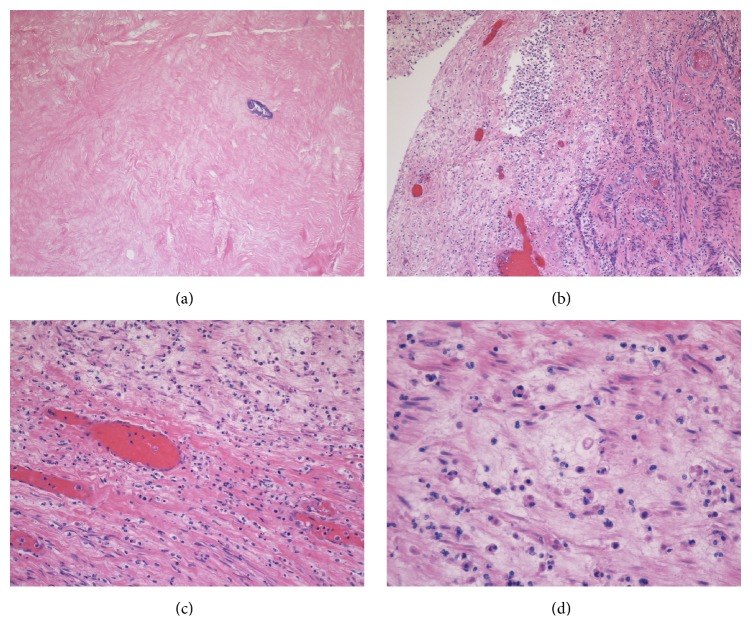
Hematoxylin and Eosin histologic appearance of the degenerating leiomyoma: (a) complete loss of nuclei from the cells on 10x magnification, (b, c) diffuse infiltrate of polymorphous neutrophils at 10x and 20x, respectively, and (d) stromal hyalinization.

Dramatic biochemical improvement occurred over the next 48 hours with CRRT following surgery. Final blood and urine cultures were negative at 48 hours. Serum creatinine decreased to 1.4 mg/dL but however rebounded slowly over the following three days and peaked at 3.1 mg/dL. Serum myoglobin reached a nadir of 380.4 ng/mL by the fourth postoperative day (POD) and steadily increased and reached a plateau of 1074 ng/mL by POD-8 (Figure 2). Clinical improvement followed with defervescence and normalization of vital signs by POD-3 and POD-5, respectively. Broad-spectrum antibiotics were discontinued at this time. The patient had normal sinus rhythm, was normotensive without any vasopressors, and was extubated uneventfully. The pathology reported a 29.4 × 20.8 × 19.7 cm large solitary leiomyoma weighing 3028 grams and showing extensive loss of nuclei from cells, stromal hyalinization, polymorphonuclear white blood cell infiltrate, and foci of calcifications reflecting various stages of early and late fibroid degenerative changes (Figure 3). Both adnexal structures showed an extensive infiltrate of polymorphonuclear cells suggestive of severe and acute inflammation.

The patient was discharged on POD-8 in a stable condition. Serum creatinine remained stable at 2.9 mg/dL, and myoglobin was 1074 ng/mL. She showed complete biochemical and clinical remission by her 4-week follow-up with a return to baseline serum creatinine (0.6 mg/dL) and a normal serum myoglobin of 25 ng/mL (Figure 2). The patient was placed on oral estradiol for hormone replacement therapy and her care was released to her primary care physician.

## 3. Discussion

Uterine fibroids can attain considerable sizes before becoming symptomatic. The compressive effects posed by a large pelvic mass may eventually lead to urinary retention, hydroureter, hydronephrosis, and potentially postrenal kidney failure. In our case, the diagnostic workup for acute renal failure and presumed septic shock identified the elevated myoglobin as an associated factor in the patient's acute renal dysfunction.

Myoglobin has a low molecular weight of 17800 daltons [3] and low protein-binding capacity [4]. It is rapidly cleared from circulation [3, 4] as it freely undergoes glomerular filtration and is reabsorbed and metabolized within the renal tubular cells [4]. Extrarenal metabolic pathways play a role as well [3, 4]. Proteolytic enzymes capable of myoglobin breakdown are present in tissue extracts of skeletal and cardiac muscles [3]. Myoglobin is primarily found within striated muscle tissue but has been detected in small amounts within uterine smooth muscle tissue [5] and may constitute up to 0.2–0.6 mg/g of uterine smooth muscle weight [2]. This smooth muscle myoglobin was found to be identical to striated myoglobin by cDNA-derived amino acid sequencing [5]. Presuming this concentration, the myoglobin content of the patient's degenerating fibroid may have approximated 1816 mg (specimen weight of 3028 g × 0.6 mg/g) but this is merely an estimate, as the specimen did not exclusively constitute smooth muscle tissue.

Elevated serum levels of myoglobin usually occur with muscle injury and hypoxia and may lead to nephrotoxicity as myoglobin is released from muscle cells [6]. It is commonly elevated in cases of rhabdomyolysis; however this typically occurs with a rise in creatinine kinase as well, a finding that did not occur in our patient. Furthermore, there was no history of acute muscle injury. Myocardial ischemia and infarction were ruled out with normal cardiac enzymes and electrocardiogram findings. Serum myoglobin levels may also be affected by hemolysis [4]; however this was ruled out in our patient as she had normal bilirubin and haptoglobin levels.

In this patient, the exact cause of suspected septic shock cannot be identified with certainty. Given that she had a recent history of worsening abdominal pain, a plausible explanation for her presentation includes systemic inflammatory response syndrome secondary to the degenerating fibroid with subsequent bacteremia and septic shock that further accentuated the degenerative inflammatory response leading to a major elevation in serum myoglobin levels and clinical deterioration. This hypothetical scenario is supported by reports of Gram-negative sepsis following uterine artery embolization for the treatment of a large solitary fibroid [7]. Our case may share the common pathway of fibroid degeneration that follows vascular compromise leading to spontaneous inflammation, bacterial seeding, and bacteremia. Although blood cultures represent an important diagnostic tool in sepsis, they only detect bacteremia in about 50% of patients who are clinically suspected to have sepsis [8]. Large studies have reported the sensitivity, specificity, and positive and negative predictive value of peripheral venipuncture blood cultures to be 95.4%, 96.9%, 85.4%, and 99.1%, respectively [9, 10]. Our patient's blood culture results were negative; nevertheless this does not rule out the possibility of bacterial sepsis and subsequent septic shock especially that the patient experienced significant clinical improvement after surgical removal of a fibroid that could have potentially been an infected one.

This patient represents a unique case of nonobstructive acute renal failure (ARF) associated with myoglobin release from a large degenerating uterine fibroid in the setting of suspected septic shock. We searched PubMed/Medline and the open access medical literature using the following MESH terms: “fibroid”, “leiomyoma”, “acute renal failure”, “sepsis”, “systemic inflammation response syndrome”, “non-obstructive”, “uterine”, “uterus”, and “gynecology”. Nonobstructive acute renal failure in light of an enlarged uterine fibroid has not been reported. Biochemical and imaging evaluation for the common causes of acute renal failure were not diagnostic in our patient. Imaging studies showed no urinary tract obstruction. A comprehensive biochemical analysis ruled out renal and prerenal causes of ARF. Intravenous hydration did not lead to any improvement in renal function or a reduction in myoglobin levels. Sepsis was unlikely with negative blood and urine cultures. Intravascular hemolysis was also ruled out. Although it is difficult to assess the effects of inflammatory mediators released from the degenerating fibroid and their potential to cause hypotension and ARF, this does not explain the elevated levels of myoglobin particularly when skeletal, cardiac, and hemoglobin sources were excluded.

The rapid posthysterectomy decrease in serum myoglobin levels is highly suggestive of the degenerating fibroid as the source of myoglobin. A steady increase in myoglobin occurred over the few postoperative days and plateaued by POD-8 at 1074 units. This is explained by the fact that myoglobin may stay longer in the circulation until renal function is restored [4]. As renal function returns to baseline, myoglobin levels continue to fall and return to normal values as noted on the patient's postoperative follow-up. Myoglobin kinetics in rhabdomyolysis-induced ARF show that the disappearance rate of myoglobin in dialyzed versus nondialyzed patients is reported to be 42.9 ± 4.0% and 39.1 ± 3.0%, from the previous day value, respectively [4]. Myoglobin in our patient did not follow this trend and this is likely secondary to the slow return of baseline renal function. There are no studies that determine the clinical effects of elevated serum myoglobin in degenerating fibroids; however studies involving rhabdomyolysis-induced renal failure suggest that a peak myoglobin level of around 4000 ng/mL might be a good predictor of ARF. Our patient's highest myoglobin level was 2140 ng/mL. This represents the peak value; however daily myoglobin levels were not obtained and thus the actual peak of serum myoglobin may have been higher.

Despite the unclear relationship between levels of myoglobinemia and development of renal failure [4], early renal protective therapies are encouraged [4, 6]. Our patient underwent CRRT as her condition was refractory to diuresis and intravenous hydration.

When considering the differential diagnosis of septic shock and acute nonobstructive renal failure in the setting of a large degenerating fibroid, the utility of serum myoglobin is important. In this setting, we suggest that any abnormal elevation in serum myoglobin should prompt the consideration of a myomectomy and/or hysterectomy especially when clinical deterioration cannot be explained otherwise. This has the potential to reverse significant morbidity and mortality. Studies that correlate the size of degenerating fibroids with serum myoglobin may shed light on the degree of renal impairment seen in such clinical settings.
